# Editor’s introduction

**DOI:** 10.1186/s40854-023-00516-z

**Published:** 2023-05-30

**Authors:** Gang Kou

**Affiliations:** Southwestern University of Finance and Economics, Chengdu, China

The 39th issue of Financial Innovation (FIN), Volume 9, No. 3 (2023) presents 18 papers contributed by authors and co-authors from Twenty-two countries and areas: Australia, Canada, Chile, China, Cyprus, Greece, Hong Kong, India, Japan, Lebanon, Netherlands, New Zealand, Norway, Pakistan, Romania, Russia, Spain, South Africa, Taiwan, Türkiye, Uzbekistan and USA. These papers can mainly be categorized into three sub themes.

## Financial economics

Kartal and Depren (2023) examines the effects of explanatory variables on domestic food prices in Turkey at different times, frequencies, and quantiles. Delgado et al. (2023) investigates the relationship between tax avoidance and earnings management in the largest five European Union economies by using artificial neural network regressions and indicates that companies in Europe’s largest economies do not evade taxes. Taha et al. (2023) examines the relationship between GDP per capita, credit for the private sector, and the ratio of export real GDP per labor force participants in selected Arab economies. Das and Gangopadhyay (2023) concludes that US food sales remained relatively immune to such massive economic and financial disruptions. Wu and Karmakar (2023) analyses the advantages of normalizing and variance stabilizing (NoVaS) method comparing with standard GARCH models and proposes a variant of the NoVaS method. Ionescu et al. (2023) addresses maximum interest for the current economy, drawing attention to the need to monitor those indicators of vulnerability that trigger risks in large urban areas and the solution to reduce these risks by developing smart cities. Akalpler (2023) reveals that public debt does not have a direct effect on the gross national product but indirectly affects total capital, consumption, investment, and public expenditure, all of which influence real gross domestic product (RGDP). Isiksal (2023) employs panel data from Central Asian States (CAS), namely, Kazakhstan, Kyrgyzstan, Tajikistan, Turkmenistan, and Uzbekistan and demonstrates an inverted U-shaped association between financial growth and natural resource rents.

## Financial markets

Narayan et al. (2023) presents an empirical method for assessing the long-term benefits of a popular portfolio investment strategy. Wang et al. (2023) develops a valuation model for a tenant’s option to renew a constant rental office lease at future market rent under an incomplete market assumption. The model and empirical results indicate that the boundaries and prices of a renewal option at future market rent can be positive or negative and depends on tenants’ private circumstances, vacancy costs, and transaction costs. Zhang et al. (2023) selects the NASDAQ CTA Artificial Intelligence and Robotics (AIRO) Index as the research target, and proposes innovative hybrid methods to forecast returns by considering its multiple structural characteristics. Kanamura (2023) proposes two new regime-switching volatility models to empirically analyze the impact of the COVID-19 pandemic on hotel stock prices in Japan compared with the US, taking into account the role of stock markets.

## Monetary

Andreadis et al. (2023) investigates the dynamic properties of the new Center for Financial Stability (CFS) Divisia monetary aggregates for the US. Alola et al. (2023) finds feedback causal effects between positive and negative shocks in monetary policy uncertainty and positive and negative shocks in the exchange rate. Reus and Sepúlveda-Hurtado (2023) presents a novel tool for generating speculative and hedging foreign exchange (FX) trading policies and finds that the volatility of currencies from emerging economies rises in comparison to currencies from developed markets. Jain et al. (2023) implies that China and Russia currency appreciation results in a trade deficit across Central Asian Economies (CAEs). Yang et al. (2023) develops a trading strategy by taking a long position on peripheral countries’ currencies and a short position on core countries’ currencies based on its findings. He et al. (2023) explores upside and downside jumps in the dynamic processes of three rates and proposes a new model which captures the risk factors to explain the exchange rate fluctuations for various economic events.

